# Phytoestrogenic Activity of Blackcurrant Anthocyanins Is Partially Mediated through Estrogen Receptor Beta

**DOI:** 10.3390/molecules23010074

**Published:** 2017-12-29

**Authors:** Naoki Nanashima, Kayo Horie, Hayato Maeda

**Affiliations:** 1 Department of Bioscience and Laboratory Medicine, Hirosaki University Graduate School of Health Sciences, 66-1 Hon-cho, Hirosaki, Aomori 036-8564, Japan; k-horie@hirosaki-u.ac.jp; 2 Faculty of Agriculture and Life Science, Hirosaki University, 3 Bunkyo-cho, Hirosaki, Aomori 036-8561, Japan; hayatosp@hirosaki-u.ac.jp

**Keywords:** anthocyanin, blackcurrant, estrogen receptor β, phytoestrogen

## Abstract

Phytoestrogens are plant compounds with estrogenic effects found in many foods. We have previously reported phytoestrogen activity of blackcurrant anthocyanins (cyanidin-3-glucoside, cyanidin-3-rutinoside, delphinidin-3-glucoside, and delphinidin-3-rutinoside) via the estrogen receptor (ER)α. In this study, we investigated the participation of ERβ in the phytoestrogen activity of these anthocyanins. Blackcurrant anthocyanin induced ERβ-mediated transcriptional activity, and the IC_50_ of ERβ was lower than that of ERα, indicating that blackcurrant anthocyanins have a higher binding affinity to ERβ. In silico docking analysis of cyanidin and delphinidin, the core portions of the compound that fits within the ligand-binding pocket of ERβ, showed that similarly to 17β-estradiol, hydrogen bonds formed with the ERβ residues Glu305, Arg346, and His475. No fitting placement of glucoside or rutinoside sugar chains within the ligand-binding pocket of ERβ-estradiol complex was detected. However, as the conformation of helices 3 and 12 in ERβ varies depending on the ligand, we suggest that the surrounding structure, including these helices, adopts a conformation capable of accommodating glucoside or rutinoside. Comparison of ERα and ERβ docking structures revealed that the selectivity for ERβ is higher than that for ERα, similar to genistein. These results show that blackcurrant anthocyanins exert phytoestrogen activity via ERβ.

## 1. Introduction

Estrogens affect the functions of organs and tissues such as bones, blood vessels, skin, and brain, and participate in the underlying mechanisms of diseases such as metabolic syndrome [1,2,3,4]. The estrogen receptor (ER) has two subtypes, ERα and ERβ. ERα is mainly present in female reproductive organs such as mammary gland and uterus, whereas ERβ is found all over the body regardless of sex. The ERβ gene was cloned in 1996 [5], and the receptor is known to be involved in several diseases such as osteoporosis [6], breast cancer [1,7,8], and obesity [9], although many functions remain unclear. Although estrogen promotes the proliferation of breast cancer cells via ERα, ERβ inhibits cell proliferation. Thus, it is known that ERβ inhibits the activity of ERα [8,10].

**Figure 1:**
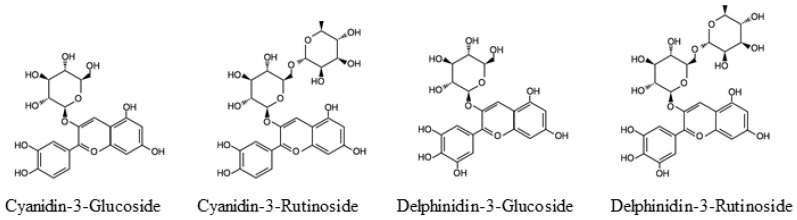
Chemical structures of anthocyanins derived from blackcurrant.

Blackcurrants (*Ribes nigrum* L.) contain high levels of flavonoids, a group of polyphenolic compounds that includes anthocyanins and flavonols. Blackcurrants are reported to contain four anthocyanins: cyanidin-3-glucoside (C3G), cyanidin-3-rutinoside (C3R), delphinidin-3-glucoside (D3G), and delphinidin-3-rutinoside (D3R) (Figure 1). D3R and C3R are anthocyanins specific to blackcurrant [11]. Blackcurrant anthocyanins are known to have some health benefits such as amelioration of obesity and inflammation and prevention of breast cancer [12,13,14].

Phytoestrogens are a chemically diverse group of plant compounds with estrogenic effects in animals and include isoflavones, lignans, coumestans, and flavonoids; they are found in many foods [15,16,17,18]. The structure of anthocyanins is similar to that of flavanones and isoflavones. Although many health benefits of blackcurrant phytochemicals have been reported, no studies have addressed their phytoestrogenic activity. In contrast, phytoestrogen activity of the anthocyanins cyanidin and delphinidin has been reported by Schmitt et al. [19]. Recently, we have reported that these anthocyanins have a phytoestrogenic effect via ERα [20], but the participation of ERβ is unknown. Liquiritigenin, genistein, and S-equol are natural ligands of ERβ [21,22,23], and are known to inhibit the proliferation of breast, prostate, and colon cancers [24]. It is becoming clear that ERβ is involved in various diseases, and it is becoming the target of pharmacological studies [25].

To improve menopause-associated symptoms, postmenopausal women may undergo hormone replacement therapy. However, when using estrogen preparations, the risk of venous thrombosis and breast cancer must also be considered. In contrast, no association of phytoestrogens with venous thrombosis has been reported, and these compounds may suppress breast cancer. Thus, phytoestrogen is considered an important alternative to estrogen preparations [26,27].

The objective of this study was to investigate whether an anthocyanin-rich blackcurrant extract (BCE) and four blackcurrant anthocyanins exert phytoestrogenic activity via ERβ. We investigated ERβ-mediated transactivation by blackcurrant anthocyanins. In addition, the binding ability of black currant anthocyanins to ERβ was determined using competition binding assays and in silico analysis of the docking of four anthocyanins to the ERβ-17β-estradiol (E2) complex. The affinity of E2 to ERβ is very similar to that of ERα, but affinity to phytoestrogens such as genistein and S-equol is high [21,23]. Therefore, based on the interaction between genistein and ERα or ERβ [28], the interaction of cyanidin with ERα or ERβ was evaluated in silico.

## 2. Results and Discussion

### 2.1. ERβ Transactivation Activity of Blackcurrant Anthocyanins

**Figure 2:**
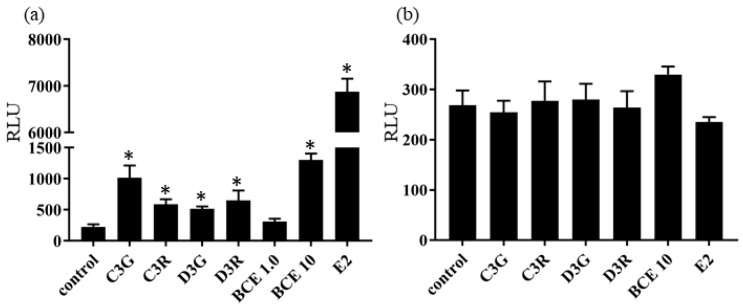
ERβ reporter assay of cells treated with 50 μM anthocyanins and 1.0 or 10.0 μg/mL blackcurrant extracts (BCE) or 100 pM 17β-estradiol (E2) in the absence (**a**) or presence (**b**) of 1.0 μM fulvestrant for 24 h. RLU, relative light units. Data are shown as the mean ± standard error of the mean of at least three independent experiments. * *p* < 0.05 vs. control.

Blackcurrant anthocyanins exhibited estrogenic activity in human ERβ reporter assays at 50.0 μM (*p* < 0.05), whereas BCE exhibited estrogenic activity at 10.0 μg/mL (*p* < 0.05), but not at 1.0 μg/mL (Figure 2a). BCE- and anthocyanin-mediated induction of estrogen response element-dependent luciferase activity was inhibited by co-treatment with 1 μM fulvestrant (Figure 2b), indicating that these effects are ERβ-mediated. These results suggest that blackcurrant anthocyanins and BCE have phytoestrogenic activity mediated via ERβ signaling.

### 2.2. Binding of Blackcurrant Anthocyanins to ERβ

**Figure 3:**
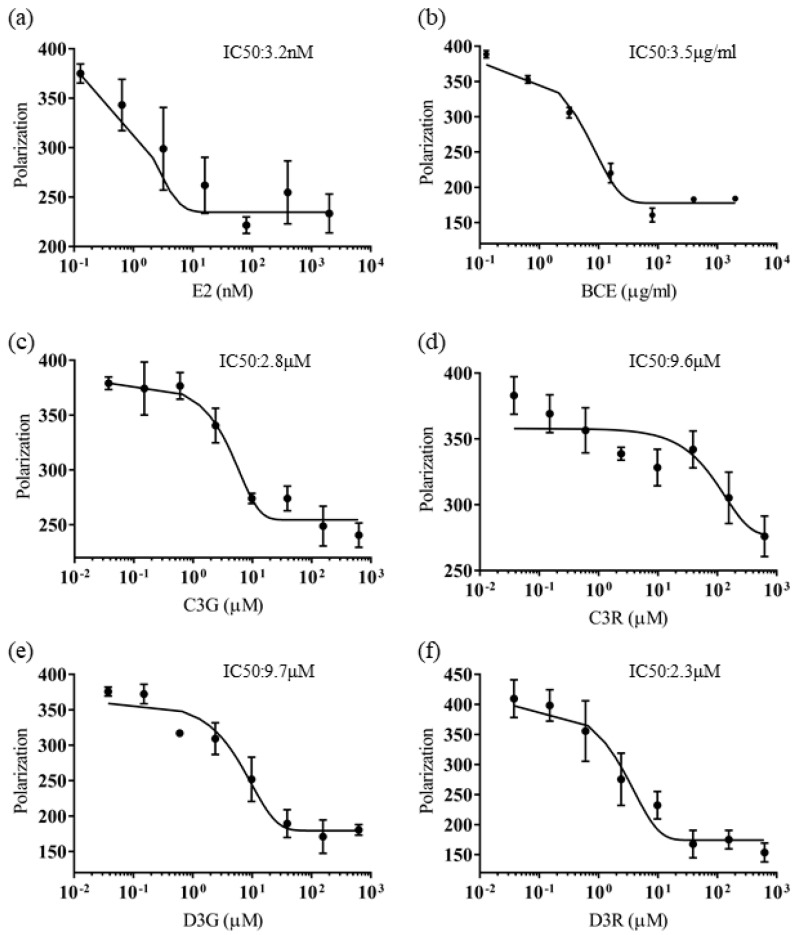
Competitive binding curves of blackcurrant anthocyanin-induced displacement of fluorescein-labeled 17β-estradiol (E2) from human ERβ. ERβ and fluorescein-labeled estradiol were incubated for 2 h with a serial dilution of (**a**) E2; (**b**) blackcurrant extract; (**c**) cyanidin-3-glucoside (C3G); (**d**) cyanidin-3-rutinoside (C3R); (**e**) delphinidin-3-glucoside (D3G); and (**f**) delphinidin-3-rutinoside (D3R) at least in triplicate. IC_50_ corresponds to the concentration of test compound inhibiting 50% of binding of 4.5 nM Fluormone ES2 Green to ERβ. Error bars represent the standard error of the mean.

We next investigated whether the phytoestrogenic activity of blackcurrant anthocyanins in vitro resulted from binding to ERβ using PolarScreen assays, and we calculated the approximate IC_50_ values. The IC_50_ of E2, BCE, C3G, C3R, D3G, and C3R was 3.2 nM, 3.5 μg/mL, 2.8 μM, 9.6 μM, 9.7 μM, and 2.3 μM, respectively (Figure 3). BCE and the four blackcurrant anthocyanins exhibited the ability to bind to ERβ. The IC_50_ of each anthocyanin was approximately 1/1000 of that of E2, which is consistent with the reported much weaker effect of phytoestrogens compared to endogenous estrogen [19,20,29]. These results suggest that blackcurrant anthocyanins have a high affinity for ERβ, similar to genistein, because the ERβ IC_50_ was lower than the ERα IC_50_ determined in our previous study [20].

### 2.3. In Silico Docking Analysis of Estradiol and ERβ

**Figure 4:**
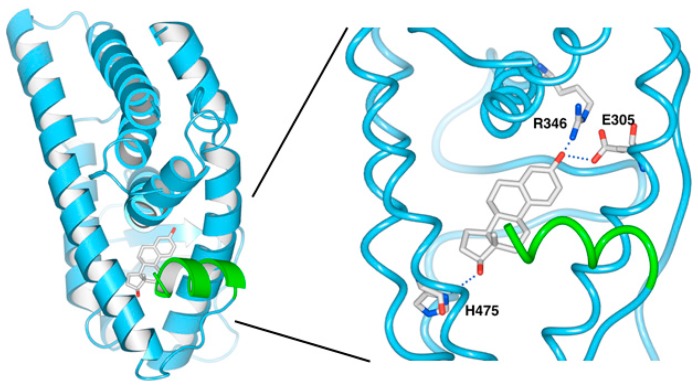
Ligand-binding pocket of the active ERβ conformation (PDB ID: 3OLS) showing interactions with 17β-estradiol (E2). Helix 12 is colored green.

The ligand-binding domain of ERβ formed a homodimer similar to that of ERα, and estradiol bound inside the ligand-binding pocket of ERβ. In the state with bound estradiol, helix 12 (green) was positioned in such a way as to close the ligand-binding pocket (Figure 4). Because the amino acid residues involved in the binding of estradiol to ERβ were not described by Mocklinghoff [30], the residues forming a hydrogen bond with estradiol were determined using the Swiss-PDB Viewer [31]. Like ERα, residues Glu305, Arg346, and His475 within the binding pocket formed a hydrogen bond with estradiol in the stereostructure (PDB ID: 3OLS) of the ERβ/estradiol complex (Figure 4).

### 2.4. In Silico Docking Analysis of C3G, C3R, D3G, or D3R and ERβ

**Figure 5:**
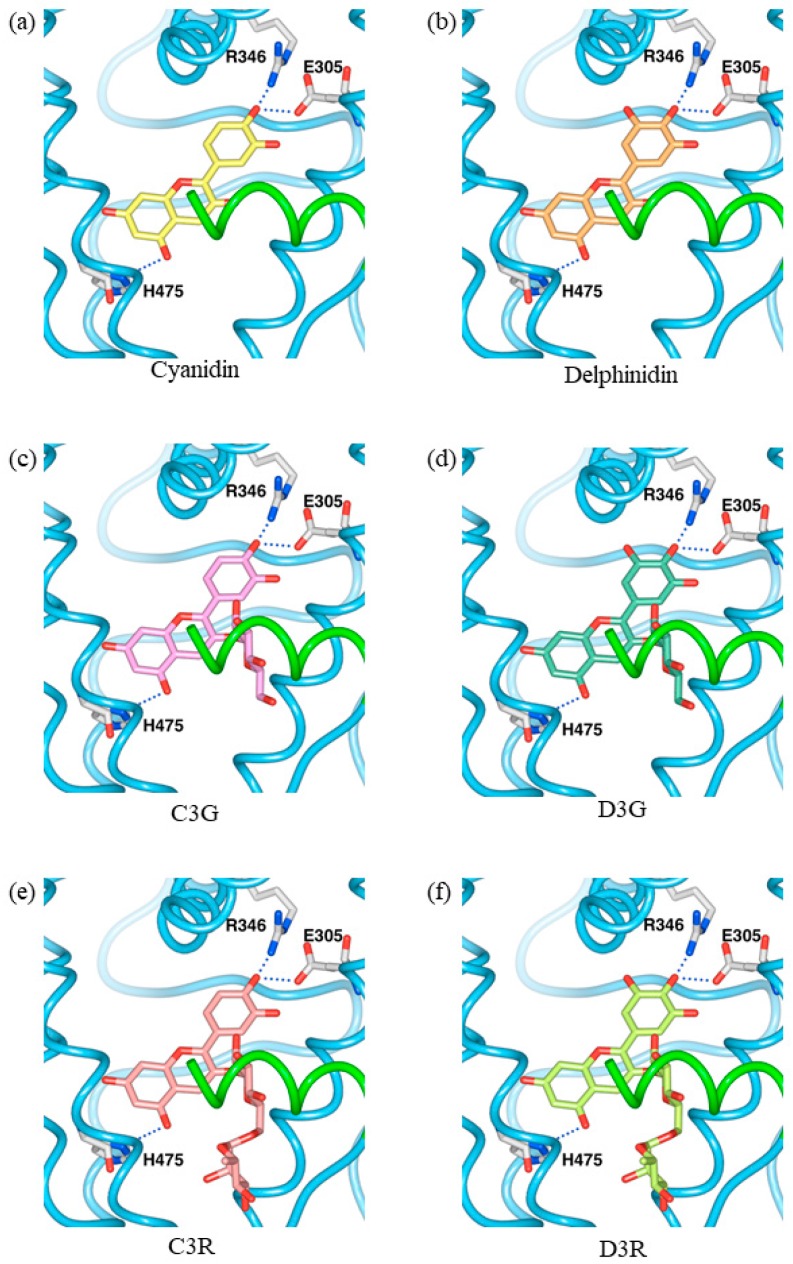
Ligand-binding pocket of the active ERβ conformation (PDB ID: 3OLS) showing interactions with (**a**) cyanidin; (**b**) delphinidin; (**c**) cyanidin-3-glucoside (C3G); (**d**) delphinidin-3-glucoside (D3G); (**e**) cyanidin-3-rutinoside (C3R) and (**f**) delphinidin-3-rutinoside (D3R). Helix 12 is colored green.

In the docking model, cyanidin and delphinidin did not collide with the amino acid residues and atoms of ERβ, and fit within the internal pocket space (Figure 5a,b). Like estradiol, the hydroxyl group at position 4 of the phenyl group of cyanidin and delphinidin formed hydrogen bonds with Glu305 and Arg346 of ERβ, and the hydroxyl group at position 5 of the benzopyrylium group formed a hydrogen bond with His475 of ERβ (Figure 5a,b). These results suggest that cyanidin and delphinidin bind inside the binding pocket of ERβ in the same arrangement as estradiol.

**Figure 6:**
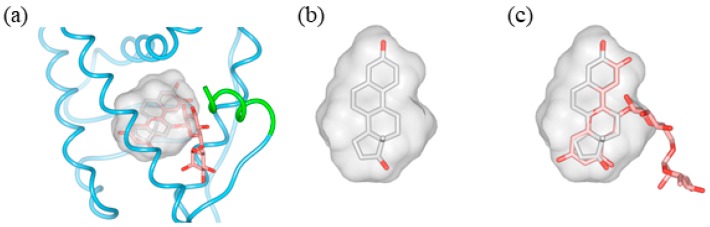
Interaction between the ligand-binding pocket of the ERβ and 17β-estradiol (E2) complex (PDB ID: 3OLS) and the sugar chain of cyanidin-3-rutinoside (C3R). (**a**) Docking model of C3R (light red) to the ERβ and estradiol complex (gray); (**b**) Surface shape of the binding site and appearance of E2; (**c**) Overlapping E2 and C3R; the sugar chain of C3R does not fit.

Based on the docking analysis of the cyanidin and delphinidin skeletons, C3G, C3R, D3G, and D3R were placed in ERβ, and the space where the glucose or rutinose at position 3 fits was investigated by rotating the bond with glucoside or rutinoside. Glucose or rutinose collided with amino acid residues present in helices 3 and 12, and an arrangement in which sugar chains fit in the space was not found (Figure 5c–f and Figure 6). These results suggest that there is not enough space inside the pocket of the ERβ-estradiol complex to bind sugar chains, which is in agreement with the report by Fan et al. [32]. However, helices 3 and 12 are known to change conformation depending on the type of ligand [33,34,35]. Therefore, if these helices have a conformation somewhat different from that of the ERβ-estradiol complex, which provides a space for accommodating sugar chains, glucoside or rutinoside may also be able to bind. Similarly, we were unable to find, using in silico docking analysis of ERα, an arrangement in which sugar chains of glucose and rutinose bind without steric hindrance, although we have previously reported that these four anthocyanins act as agonists [20]. In ERβ, it was also suggested that helices 3 and 12 form an appropriate conformation for four kinds of anthocyanins, thereby indicating that helix 12 adopts an agonist-like arrangement.

### 2.5. Differences in Anthocyanin Binding to ERα and ERβ

**Figure 7:**
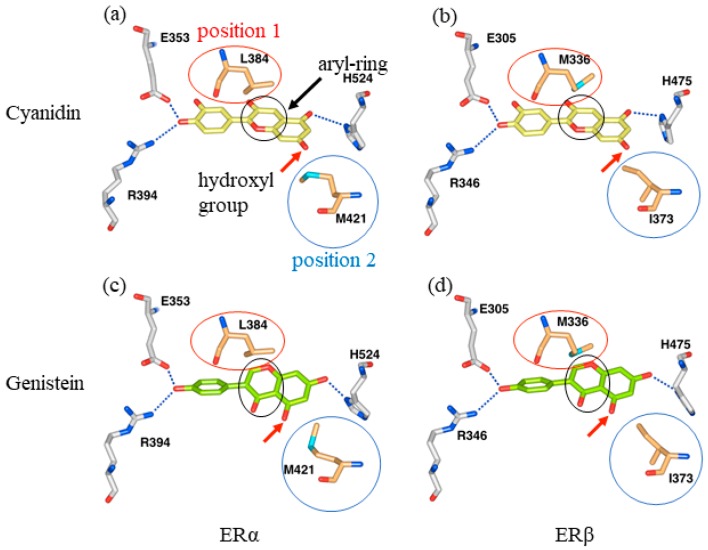
Difference in binding affinity of cyanidin to ERα and ERβ. Hydrogen bonds of each compound and ERs are indicated as blue dotted lines. Red, blue, and black circles indicate position 1, position 2, and aryl ring, respectively. Red arrows indicate hydroxyl groups. (**a**) Interaction of cyanidin with ERα; (**b**) cyanidin with ERβ; (**c**) genistein with ERα; and (**d**) genistein with ERβ.

Manas et al. have determined the conformation of the genistein/ERα and genistein/ERβ complexes (PDB ID: 1X7R and 1X7J) and reported the selectivity factor of genistein to ERβ [28]. To investigate the differences in anthocyanin interaction with ERα and ERβ, we used ERα/cyanidin and ERβ/cyanidin complex models, and each ER residue located within 5.0 Å from each atom of cyanidin was determined using the Waals software. Nineteen residues were detected, and the only two residues different between ERα and ERβ were ERα Leu384 and ERβ Met336, and ERα Met421 and ERβ Ile373 (Figure 7a,b and Table 1). These differences are consistent with those reported in the genistein and ERα and ERβ binding pockets [28]. Hydrogen bonds form between cyanidin and Glu305, Arg346, and His475 of ERβ, and these residues are conserved in ERα (Figure 7a,b and Table 1).

Hydrophobic interactions of Ile373 in ERβ, in addition to those of Ala302 and Phe356, corresponded to the interactions of Ala350 and Phe404 in ERα, which was inferred from the complex containing cyanidin (Table 1). In this study, the positions of ERα Leu384 and ERβ Met336 were named position 1, and the positions of ERα Met421 and ERβ Ile373 were named position 2 (Figure 7).

We observed stabilization of the protein structure in an interaction between methionine and aromatic rings, called the methionine-aromatic interaction, and selectivity to ERβ in compounds having an aryl aromatic ring positioned in the B-ring of genistein (Figure 7c,d), and thus ERβ Met336 is estimated to have a more favorable interaction with the aryl group of genistein than ERα Leu384 [28]. Therefore, this interaction is considered to underlie the selectivity of genistein for ERβ rather than ERα. Cyanidin and delphinidin have aryl groups at positions corresponding to the B-ring of genistein (Figure 1 and Figure 7a,b). Based on the report of Manas et al. we suggest that cyanidin and delphinidin can also interact more favorably with ERβ Met336 compared to ERα Leu384, similar to genistein [28].

There is a hydroxyl group (5-OH) at position 5 of genistein near position 2 (Figure 7c,d). The side chain of Met421 in the ERα/genistein complex (PDB ID: 1X7R) adopts a rotamer whose lone pair of sulfur atoms avoids the oxygen atom of 5-OH of genistein. Furthermore, it is different from the rotamer of the side chain of Met421 of the ERα/estradiol complex (PDB ID: 1ERE) [30]. It is also known that dimethylsulfide clearly repels hydroxyl groups and that propane attracts weakly at an angle in which lone pairs of electrons face each other [28].

The hydroxyl group of position 7 of cyanidin is in the vicinity of ERα Met421 and ERβ Ile373 (Figure 7a,b). The analysis of the genistein complex suggests that the hydroxyl group at this position may repel ERα Met421 when binding to ERα (Figure 7c). In contrast, we suggest that ERβ does not repel ERβ Ile373, and the side chain of Ile373 and the carbon atoms at positions 6 and 7 may form a hydrophobic interaction (Figure 7a,b and Table 1). Therefore, ERβ Ile373 seems to be more accommodating to cyanidin and delphinidin structures than ERα Met421.

Given that estrogen levels decrease after menopause, dietary phytoestrogen may alleviate postmenopausal health concerns related to skin, bone, and cardiovascular heath [36,37,38,39]. In addition, it is known that E2 also affects male diseases such as benign prostatic hyperplasia and prostate cancer [40,41]. In particular, ERβ is expressed regardless of sex, and thus it is important to consider this receptor as a therapeutic target [25]. Furthermore, we previously orally administered BCE to female rats aged 3 weeks, and showed that BCE also had phytoestrogenic activity also in vivo [20]. We thus predict that as phytoestrogens, blackcurrant anthocyanins have many biological activities.

## 3. Materials and Methods

### 3.1. Materials

The BCE powder, CaNZac-35, was purchased from Koyo Mercantile (Tokyo, Japan). BCE contains high concentrations of polyphenols (37.6 g/100 g BCE) and anthocyanins (38.0 g/100 g BCE) [20]. C3G, C3R, D3G, and D3R (see Figure 1 for chemical structures) were purchased from Nagara Science (Gifu, Japan). E2 and fulvestrant (ICI 182,780) were purchased from Sigma-Aldrich (St. Louis, MO, USA).

### 3.2. ER Transactivation Assays

To assess the activation of human ERβ, nuclear receptor transactivation assay kits were obtained from Indigo Biosciences (State College, PA, USA). Briefly, the test compounds were prepared and diluted in a medium provided by the manufacturer. The cell recovery medium provided in the assay kit was thawed, warmed to 37 °C, and added to the frozen reporter cells. The cell suspension (100 μL) was dispensed into the wells of a 96-well assay plate and the test compounds (100 μL) were added to the cells at the indicated concentrations and incubated for 24 h. Luciferase activity was quantified using a TriStar LB941 multimode plate-reader (Berthold Technologies, Bad Wildbad, Germany).

### 3.3. Competitive Binding Assays

Competitive binding assays were performed using the PolarScreen ERβ Competitive Binding Assay Kit Green (Life Technologies, Carlsbad, CA, USA) according to the manufacturer’s protocol. Recombinant human ERβ (23 nM) and 4.5 nM Fluormone ES2 Green (fluorescently labeled estradiol) were incubated for 2 h with the test compounds. Fluorescence polarization was measured using a Flex Station 3 (Molecular Devices, Sunnyvale, CA, USA). Approximate IC_50_ values, which indicate the ligand concentration that yields 50% inhibition of Fluormone ES2 Green, were determined from competitive binding curves generated using GraphPad Prism ver. 7.03 for Windows (GraphPad Software, San Diego, CA, USA).

### 3.4. Molecular Docking Simulations

In silico docking analysis was performed to investigate the interactions between blackcurrant anthocyanins and ERβ. The interaction between E2 and ERβ was used as positive control. The steric structures of C3G and C3R were obtained from the ZINC (http://zinc.docking.org) compound database (AC4097706 and AC4097715, respectively). D3G and D3R steric structure models were constructed using MarvinSketch (ChemAxon http://www.chemaxon.com/products/marvin/) based on the structures of C3G and C3R, respectively. Docking models based on the X-ray crystal structure of human ERβ with E2 were obtained from the Protein Data Bank (PDB) (http://www.rcsb.org/pdb/) (PDB ID: 3OLS) [35], which enabled analysis of docking to the active type (with E2) of ER. The steric structures of anthocyanins were fitted to the ER steric structure by superimposition on the molecular frame structure of E2 using Waals (Altif Laboratories, Tokyo, Japan). Hydrogen bonding and atomic interactions were determined using Swiss-Pdb Viewer programs available at http://swissmodel.expasy.org/. These analyses were performed at Altif Labs.

### 3.5. Statistical Analysis

Results are expressed as the mean ± standard error of the mean (SEM) of at least three independent experiments. Statistical analyses were performed using BellCurve for Excel ver. 2.13 software (Social Survey Research Information, Tokyo, Japan) and Kruskal-Wallis analysis with the Steel post-hoc test; *p* < 0.05 was considered to indicate statistical significance.

## 4. Conclusions

We investigated the possibility of blackcurrant anthocyanins binding to ERβ. The results show that these anthocyanins induced ERβ transcriptional activity, and that the IC_50_ was smaller for ERβ than for ERα. Consistent with these results, the affinity for ERβ was higher than that for ERα. In the structure of the ERβ/estradiol complex, some steric hindrance was found between sugar chain atoms and helices 3 and 12. However, as the conformation of these helices varies dynamically, we suggest that when each of the four blackcurrant anthocyanins bind to ERβ, they adopt a conformation suitable for accommodating glucoside or rutinoside. These results reveal that blackcurrant anthocyanins have phytoestrogen activity via ERβ. Therefore, blackcurrant anthocyanins may be effective for improvement of various senile-stage disorders known to be associated with ERβ, such as menopausal disorder and breast cancer.

## Figures and Tables

**Table 1:** Comparison of predicted interactions between cyanidin and ERα or ERβ.

Amino Acid Residue		Interaction with Cyanidin	
ERα	ERβ	Common to ERα & ERβ	ERβ only
Ala350	Ala302	hydrophobic interaction	
Glu353	Glu305	hydrogen bond	-
Leu384	Met336		interaction with aryl ring (position 1)
Arg394	Arg346	hydrogen bond	-
Phe404	Phe356	hydrophobic interaction	-
Met421	Ile373	-	hydrophobic interaction
			interaction with hydroxyl group (position 2)
His524	His475	hydrogen bond	-

Each ER amino acid residue is shown located within 5.0 Å from each atom of cyanidin. -: none.
